# Nucleotide Identification in DNA Using Dielectrophoresis Spectroscopy

**DOI:** 10.3390/mi11010039

**Published:** 2019-12-28

**Authors:** Fleming Dackson Gudagunti, Logeeshan Velmanickam, Dharmakeerthi Nawarathna, Ivan T. Lima

**Affiliations:** Department of Electrical and Computer Engineering, North Dakota State University, Fargo, ND 58102, USA; Fleming.Gudagunti@ndsu.edu (F.D.G.); Logeeshan.Velmanicka@ndus.edu (L.V.); Dharmakeerthi.Nawara@ndsu.edu (D.N.)

**Keywords:** single nucleotide polymorphism, dielectrophoresis, spectroscopy, bioelectronics

## Abstract

We show that negative dielectrophoresis (DEP) spectroscopy is an effective transduction mechanism of a biosensor for the detection of single nucleotide polymorphism (SNP) in a short DNA strand. We observed a frequency dependence of the negative DEP force applied by interdigitated electrodes to polystyrene microspheres (PM) with respect to changes in both the last and the second-to-last nucleotides of a single-strand DNA bound to the PM. The drift velocity of PM functionalized to single-strand DNA, which is proportional to the DEP force, was measured at the frequency range from 0.5 MHz to 2 MHz. The drift velocity was calculated using a custom-made automated software using real time image processing technique. This technology for SNP genotyping has the potential to be used in the diagnosis and the identification of genetic variants associated with diseases.

## 1. Introduction

Genetic markers are used to follow the inheritance patterns of chromosomal regions from generation to generation and are used in identifying the genetic variants associated with human diseases [1]. The most common genetic variation is single nucleotide polymorphism (SNP), which is due to the differences of a single base substitution. In every DNA sequence, SNP represents a difference in a single nucleotide [2]. SNP may replace a single nucleotide with another nucleotide [3]. Previous research showed that SNP predicts an individual’s risk of developing certain diseases such as cardiovascular disease, type 2 diabetes mellitus [4], autoimmune disease, Alzheimer’s disease [5], cancer, and an individual’s response to certain drugs [6,7,8,9,10]. These small differences can be used to track an individual’s susceptibility to environmental factors such as toxins [11]. Since SNPs are stable over generations, they are excellent genetic markers. It is important to explore the role of SNPs in the genetic analysis of diseases, as they would enable the identification of complex diseases and genetic disorders [12]. Even though SNPs have been shown to be an important factor in genetic variation [13,14], detecting SNPs is still expensive and time-consuming with existing techniques.

SNP genotyping can be performed with DNA sequencing methods [15]. Mapping and assembly with quality (MAQ) maps shotgun reads can build the assemblies by using value-based scores to derive genotype calls of the consensus sequence of a diploid genome. This technique is based on the Bayesian statistical model that includes error probability and mapping qualities from the quality scores of the sequence. Although minimum order quality (MOQ) [16] is efficient and highly sensitive, the high probability of sequencing errors in this method makes it less reliable than other methods.

The Short Oligonucleotide Analysis Program (SOAP) package is a resequencing tool that compares raw sequencing reads with the reference genome to calculate the probability of each possible genotype [17]. This method incorporates data quality, alignment, and recurring experimental errors, making this method complex with large acquisition time.

TaqMan PCR is a fast and reliable tool for genotyping. There are few applied biosystem instruments such as real time PCR available for the processing of TaqMan SNP genotyping assays. The endpoint read can be performed on applied biosystems such as real time PCR. The results from this method are highly accurate and reproducible, although the biosystem involved is expensive and time consuming. The amplification of the alleles is done using two pairs of primers. This process involves overlapping of the primer pairs so that it matches the pairs but not to the alternative allele for the SNP [15,16,17,18]. The conventional techniques for SNP detection rely on gel electrophoresis for the fragment analysis [19,20,21]. Several techniques include oligonucleotide ligation [22,23,24], extension of primers [25,26,27], allele-specific DNA hybridization [28], or electrochemical typing [29,30].

To address the need for newer technologies for SNP detection that could potentially simplify at least one of the steps in the SNP detection, we developed a rapid SNP genotyping method based on dielectrophoresis (DEP) spectroscopy that does not rely on fluorescence. DEP spectroscopy has the potential to be a cost-effective transduction mechanism for SNP detection in short single-stranded DNA (ssDNA), since it replaces the fluorescence tagging that is used in the last step of most of the existing techniques. DEP has been widely used for manipulation, separation, and characterization of cells, DNA, viruses, and colloid particles (at both micro and nano scales) in various microfluidic platforms, since it has numerous unique advantages [31].

In a previous study, we showed that DEP spectroscopy was an effective method for the detection of the pancreatic cancer biomarker CA 19-9 in serum [32,33]. In this study, we show that this method, which does not rely on fluorescence labeling, can also be used in SNP genotyping in a short DNA strand, and it also has the potential to be used in sequencing.

## 2. Theory

Dielectrophoresis (DEP) [34] is a physical phenomenon in which the coulomb force acts on electrically polarized dielectric particles in a non-uniform electric field. Since the electric field is non-uniform, the dielectric particles can experience translational force towards the region with higher electric field if they are more polarizable than the medium. This phenomenon is denoted positive DEP. If the medium is more polarizable than the dielectric particle, the dielectric particles suffer a net force towards the region with lower electric field. This phenomenon is denoted negative DEP [34,35,36,37]. Since the DEP force is strongly dependent on the characteristics of the particles and the molecules bound to their surfaces, DEP has been used to isolate, concentrate, or separate different types of target particles [38,39,40,41,42,43]. However, the physics behind the frequency-dependence of the DEP force on the molecular structures is still not well understood [40,44,45].

## 3. Materials and Methods

### 3.1. Sample Preparation

The sample preparation consisted of the following steps:

Step 1: Streptavidin attachment to biotinylated polystyrene microspheres (PM). Biotinylated PM with 750 nm diameter were purchased from Spherotech Inc (Lake Forest, IL, USA). The first step in the preparation of samples consisted of binding the biotinylated PM with the antigen Streptavidin purchased from Vector Labs Inc. Biotin acted as a conjugate to the protein Streptavidin, and they formed a strong bond with very high affinity. This process was done first by a 3 µL Streptavidin solution into a 10 µL biotinylated PM solution at 70 °F in a centrifuge tube to have 100% binding [46], according to the manufacturer’s recommendation. This procedure recommended by the manufacturer might not produce 100% binding. However, inaccuracies in the protocol recommended by the manufacturer should not have affected the fact that the DEP force depends on both the frequency and the nucleotides near the end of a DNA sequence, since the same protocol was used in all the experiments with different DNA sequences, and four batches were prepared for each sequence to validate these results. The total volume was set to 400 µL by adding 0.01× phosphate-buffered saline (PBS) solution with conductivity 0.01 S/m. Then, the sample was uniformly mixed using a vortex machine and left on a shaker for 20 min for the Streptavidin–biotin binding process. After 20 min, the tube was centrifuged at 5000 rpm for 14 min to remove the unbound Streptavidin molecules and the buffer.

Step 2: Biotinylated DNA attachment to the biotinylated PM + Streptavidin. Each Streptavidin molecule can bind up to 4 biotin molecules. One binding site of each Streptavidin molecule was used to bind that Streptavidin molecule with the PM. The remaining three Streptavidin binding sites bound with three biotinylated DNA. First, 3 µL of biotinylated DNA purchased from The Midland Certified Reagent Company was added into 397 µL of 0.01× Tris-EDTA to have 100% binding of the biotinylated DNA with all the Streptavidin molecule binding sites, according to the manufacturer’s requirement. Then, this sample was added into the solution with Streptavidin–biotin PM and uniformly mixed using a vortex machine. After that, the sample was kept on a shaker for 20 min at 70 °F for the biotinylated DNA to bind with the Streptavidin molecules of the PM. After 20 min, the tube was centrifuged at 5000 rpm for 14 min to remove the unbound DNA antibody molecules and the buffer.

Step 3: Preparation of the solution for the experiments. First, 200 µL of 0.01× TE buffer was added to the centrifuge tube and mixed uniformly. Then, 10 μL of this sample solution was pipetted on to the microelectrodes and used for each experiment.

The ssDNA sequences with difference in last nucleotide were:

5′-(biotin) TGTTGTGCG*A*-3′
5′-(biotin) TGTTGTGCG*T*-3′
5′-(biotin) TGTTGTGCG*G*-3′
5′-(biotin) TGTTGTGCG*C*-3′

The ssDNA sequences with difference in the second-to-last nucleotide were:

5′-(biotin) TGTTGTGC*A*C-3′
5′-(biotin) TGTTGTGC*T*C-3′
5′-(biotin) TGTTGTGC*C*C-3′

### 3.2. DEP Spectroscopy: Hardware and Software

**Figure 1:**
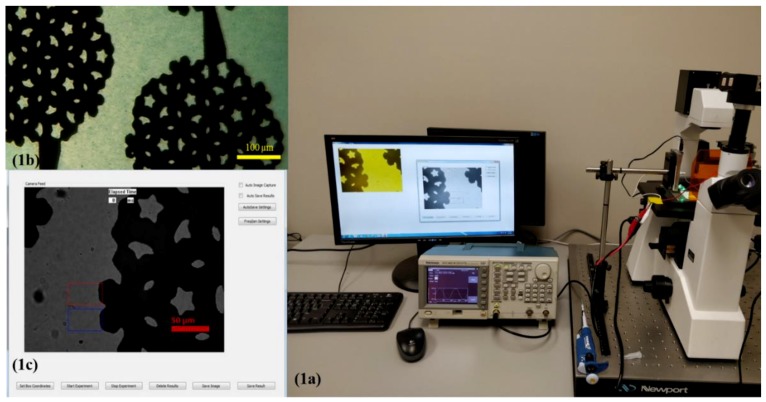
Single nucleotide polymorphism (SNP) sensor prototype: (**a**) experimental setup, (**b**) interdigitated electrode used in the experiments. The electrode is visible as the darker region in the picture. The yellow scale bar indicates 100 µm. Multi-colored hollow rectangles depict the regions of interest for measurements and analysis. (**c**) Interface of Microsoft Windows application for dielectrophoresis (DEP) spectroscopy. The red scale bar indicates 50 µm.

To measure the DEP spectrum to characterize the nucleotide in ssDNA, we used the same experimental setup that we used to measure the concentration of CA 19-9 in spiked serum [33]. We developed a software package using Microsoft foundation classes in visual C++ in the Windows operating system. The software application controlled a USB video class (UVC) standard compliant ProScope Digital Microscope 5 MP Camera (ProScope, Wilsonville, OR, USA) and a Tektronix AFG series function generator (Tektronix Beaverton, Beaverton, OR, USA). With the application controlling the function generator, the frequency was swept to measure the DEP force applied to functionalized PM as a function of the frequency. The experimental set up is shown in Figure 1. The microscope camera extracted the pixel information of the live video using a real-time image processing algorithm. The region of interest to observe the DEP effect was set by placing rectangular boxes (as shown in Figure 1c) in which the pixel and the color information were extracted, and with this data, the drift velocity due to DEP force as a function of frequency of the electric field was calculated using the algorithm.

The direction of movement for the dielectric particle due to DEP depended upon the relative polarizability of the particle and the medium as well as on the presence of a large gradient of the electric field intensity produced by electrodes [47]. In the experiments, we used the interdigitated electrodes shown in Figure 1b. The electrode was fabricated on a commercially available glass wafer with standard fabrication procedures including photolithography, metal sputtering, and lift-off procedures using 1000 Å thick gold film in the microfabrication facilities at North Dakota State University. Using COMSOL, we verified the maximum electric field of the electrode was 1.8 × 10^4^ V/m and the gradient of the electric field intensity was as high as 3 × 10^12^ V^2^/m^3^.

### 3.3. Spectrum Measurement

The drift velocity was measured from the point of application of positive DEP to the negative DEP at the applied frequency. Positive DEP was used to attract the PM to the convex edge of the electrodes. That was done through the application of a low frequency such as 10 kHz to the electrodes. Negative DEP was used to repel the PM from the convex edge of the electrodes to enable drift velocity measurements, which were produced with frequencies on the order of hundreds or thousands of kHz. This process enabled an indirect measurement of the negative DEP force, since the DEP force was proportional to the drift velocity due to the viscosity of the solution. The custom-made software was programmed to sweep a set of alternating frequency generating positive and negative DEP. The experiments were conducted with frequencies from 0.5 MHz to 2 MHz in linear steps with 10 V peak-to-peak.

A preliminary set of experiments were conducted to determine the choice of time intervals for the application of negative and positive DEP. The software automatically switched the frequency to a higher value after 1000 ms, producing negative DEP effect for 40 ms. The cycle is repeated with the application of positive DEP and the following frequency that produced negative DEP until the stop frequency is reached. The system acquired the frames at 0 ms and at 40 ms after the application of a frequency that produced negative DEP to measure the average drift velocity of the PM. The function generator used in the experiments was a Tektronix AFG series that was controlled by the computer via USB port. The negative DEP spectrum was measured indirectly through the average drift velocity of the PM as a function of the frequency, since the drift velocity is proportional to the negative DEP force.

## 4. Results

**Figure 2:**
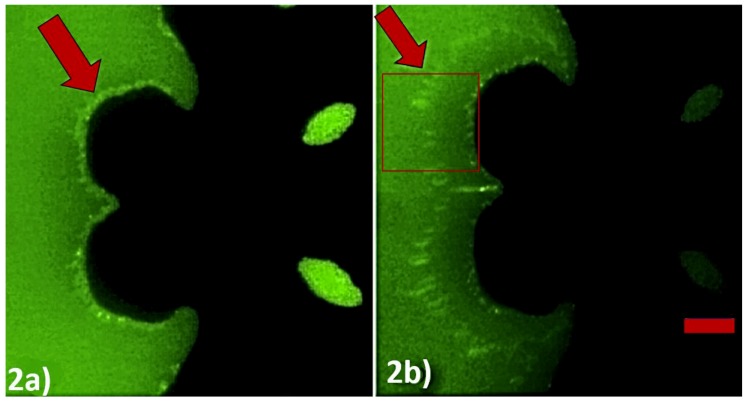
Experimental demonstration of negative DEP effect through time-lapse images captured through DEP spectroscopy application. The electric field was changed from 10 kHz to 0.5 MHz with 10 V peak-to-peak at *t* = 0 ms. (**a**) *t* = 0 ms and (**b**) *t* = 40 ms. The ssDNA sequence used for this was 5′-(biotin) TGTTGTGCGA-3′. The interdigitated electrode is visible as the darker region in the picture. The bright layer visible on the edge of the electrode is formed by the accumulation of PMs. The scale bar indicates 25 µm. The rectangular box depicts the region of interest processed to extract the position of the PM band as a function of the time.

We validated our method to detect SNPs using DEP spectroscopy experiments with our custom-made image processing software for observation and data acquisition. The principle of operation of our method is shown in Figure 2. The initial frequency was set to 10 kHz to produce positive DEP (< 50 kHz), and the sweeping frequency from 0.5 MHz to 2 MHz produced negative DEP (> 250 kHz). Figure 2a clearly shows the application of positive DEP, which concentrates PMs on the edge of the convex regions of the electrodes up to 0 ms, when negative DEP was applied. Figure 2b shows the position of the PM band while it was being repelled away from the convex regions of the electrode due to negative DEP after 40 ms. We developed a system that can calculate the drift velocity of the PM layer to indirectly measure the negative DEP force as a function of the applied frequency.

**Figure 3:**
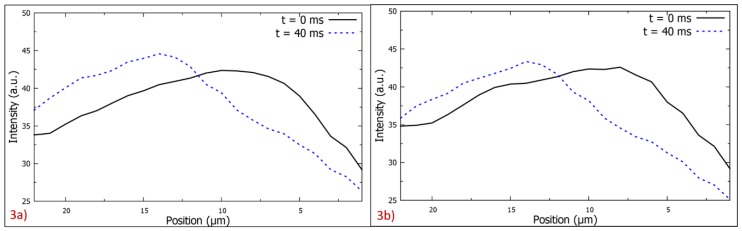
Light intensity as a function of the pixel position at *t* = 0 ms, when negative DEP was applied, and at *t* = 40 ms (**a**) for the ssDNA sequence 5′-(biotin) TGTTGTGCGG-3′, which corresponded to the results shown in Figure 2, and (**b**) for the ssDNA sequence 5′-(biotin) TGTTGTGCGA-3′ in 10 µL at the frequency 0.5 MHz. The convex edge of the electrode is located on the right side of the polystyrene microspheres (PM) layer, as shown in Figure 2.

The region of interest, which is depicted as the rectangular box in the Figure 1c, was processed to extract the position of the PM band as a function of the time while a frequency that produced negative DEP was applied. Figure 3 represents the light intensity of the images captured shortly after the application of negative DEP and 40 ms later. The average location of the PM was the center of mass $x_{\mathrm{CM}}$ of the light intensity curves. The change in the center of mass of the curves resulted from the movement of the PM band due to the application of a frequency that produced negative DEP. The center of mass was an effective way to measure the location of the PM band to determine the velocity of the PM band. The formula used to calculate the center of mass of the light intensity curve is given by:

$$x_{\mathrm{CM}}=\frac{\sum_{i=1}^{N}I_{i}x_{i}}{\sum_{i=1}^{N}I_{i}}$$

Where $I_{i}$ is the value of the light intensity at the distance $x_{i}$ from the convex edge of the electrode. Only the region whose intensity exceeded two-thirds of the peak intensity was included in the calculation of the center of mass.

**Figure 4:**
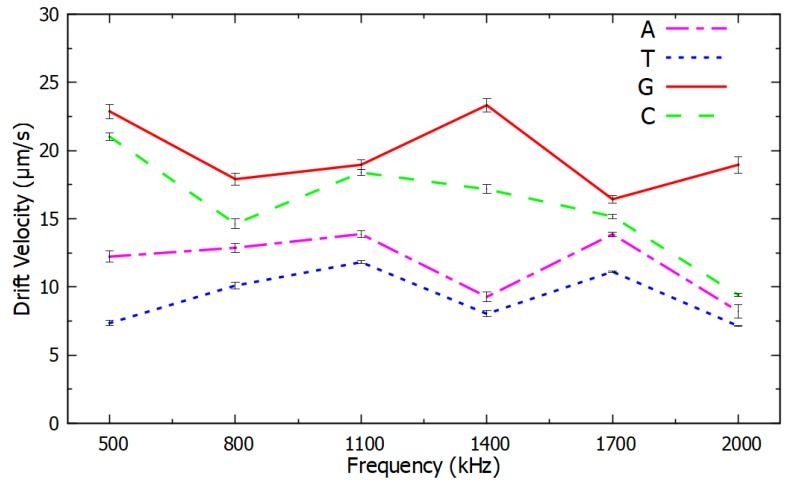
Negative DEP spectra for different nucleotides at the end of ssDNA sequences averaged over six measurements per frequency. All the other nucleotides were the same in the ssDNA sequences. The error bars show the confidence interval of the average value.

**Figure 5:**
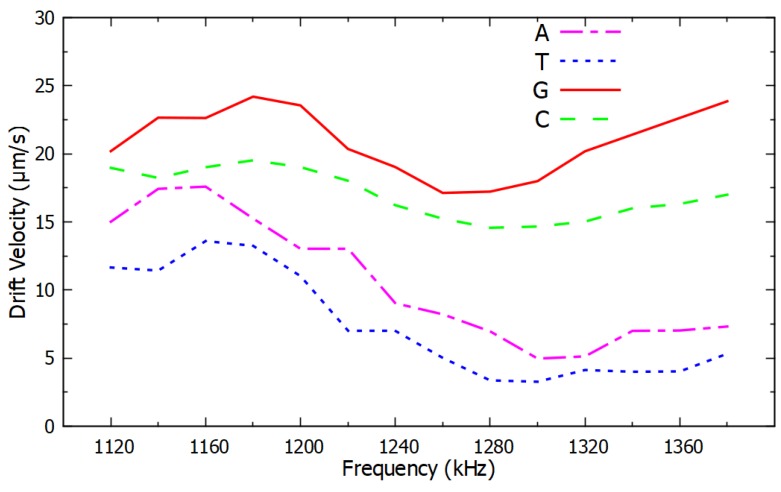
High resolution negative DEP spectra from 1120 kHz to 1380 kHz for the same ssDNA sequences used in Figure 4.

**Figure 6:**
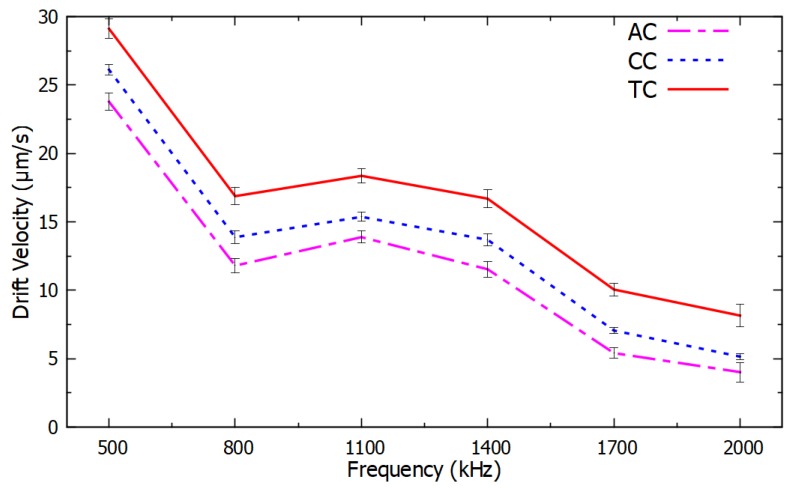
Negative DEP spectra for different nucleotides adjacent to the last nucleotide of ssDNA sequences averaged over six measurements per frequency. All the other nucleotides were the same in the ssDNA sequences. The error bars show the confidence interval of the average value.

**Figure 7:**
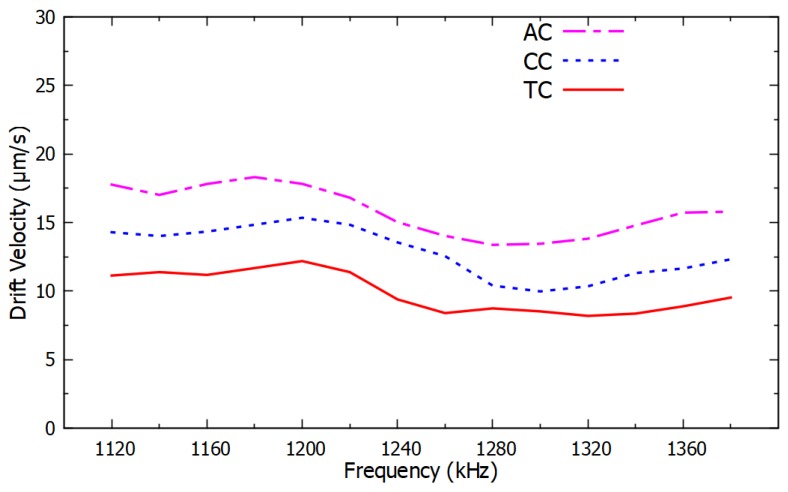
High resolution negative DEP spectra from 1120 kHz to 1380 kHz for the same ssDNA sequences used in Figure 6.

A relationship was observed between the drift velocity of the PM functionalized with ssDNA, which was proportional to the negative DEP force, as a function of frequency and the type of nucleotide in the last and in the second-to-last nucleotide. The ssDNA sequence considered in these experiments was 5′-(biotin) TGTTGTGCGA-3′, and its variations in the last and in the second-to-last nucleotide are listed in Section 3. A clear dependence was observed between the negative DEP spectrum and the nucleotide sequence. The resulting DEP spectra are shown in Figure 4 and Figure 5 for changes in the last nucleotide and Figure 6 and Figure 7 for changes in the second-to-last nucleotide. Each DEP spectrum curve shown in these figures required only 68 s to be obtained during the experiments. Figure 5 shows a higher resolution spectrum for change in last nucleotide, and Figure 7 shows the high-resolution spectrum for change in the second-to-last nucleotide over the same frequency range shown in Figure 5.

The difference in the negative DEP spectra shown in Figure 4, Figure 5, Figure 6 and Figure 7 resulted only from changes in a single nucleotide of the ssDNA sequence. No other parameters and conditions were changed throughout the experiments. The difference in the nucleotides near the end of the ssDNA bound to the PM caused a sufficiently different polarizability of the PM to affect the real part of the Clausius–Mossotti factor that determines the DEP force [35,36]. The narrow error bars shown with plus and minus one standard deviation of the average of six measurements in Figure 4 and Figure 6 are indications of the high accuracy and the repeatability of the measurements. Since there was no overlap in the confidence intervals of different curves in Figure 4 and Figure 6, these results are sufficient to determine the type of the last and the second-to-last nucleotide of these ssDNA sequences. Therefore, these results demonstrate the potential use of this method as a novel transduction mechanism for SNP detection in the last and in the second-to-last nucleotides in ssDNA sequences. The DEP spectra shown in Figure 4, Figure 5, Figure 6 and Figure 7 can be used as the calibration curve for the SNP detection in the sequences investigated. The results with guanine in the second-to-last position of the ssDNA sequence were not shown in Figure 6 and Figure 7 because they were undermined by the hairpin effect that led to clustering of the PM, which is a limitation of this method.

## 5. Conclusions

We demonstrated that the negative DEP spectroscopy method is an effective transduction method to accurately detect SNPs in short DNA strands. The negative DEP spectrum was measured using a custom-made real time image processing technique to detect the drift velocity of PM bound to ssDNA in microstructured electrodes, since the drift velocity is proportional to the DEP force. The frequency-dependent velocity of repulsion due to negative DEP on a set of PMs bound to ssDNA that we measured using image processing had a strong dependence on the last and on the second-to-last nucleotides in the ssDNA sequences that we investigated. This technique does not require the use of fluorescent labels, which eliminates the washing step of unbound fluorescent molecules in the preparation process and consequently does not require a careful calibration of the light source intensity and the photodetector sensitivity. The shape of the negative DEP spectrum curve used in our method for SNP genotyping is a measurement that does not depend on the light intensity, the number of PM, or the sensitivity of the microscope camera. The DEP force on dielectric microspheres depends on the polarizability of the microspheres, the polarizability of the ssDNA bound to the microspheres, and the polarizability of the molecules in the solution. The polarization mechanism of DNA is still not fully understood [36,48]. We are currently improving this method by designing a microstructured electrode array that is optimized for negative DEP spectroscopy. We plan to investigate the practical application of this method to detect SNPs by studying a large number of ssDNA with different nucleotide sequences and different lengths to assess the effectiveness of this method in SNP genotyping and DNA sequencing for clinical applications.

## 6. Patent

I. T. Lima Jr., D. Nawarathna, F. D. Gudagunti, and L. Velmanickam, “Method for Detecting and Quantifying Biological Molecules using Dielectrophoresis,” Patent Application No. US 2019/0234,902, August 1, 2019.
